# Comparison of Physicochemical Properties of Two Types of Polyepichlorohydrin-Based Anion Exchange Membranes for Reverse Electrodialysis

**DOI:** 10.3390/membranes12030257

**Published:** 2022-02-24

**Authors:** Ezgi Karakoç, Enver Güler

**Affiliations:** Department of Chemical Engineering, Atılım University, Ankara 06830, Turkey; ezgi.kkoc@gmail.com

**Keywords:** anion-exchange membrane, polyepichlorohydrin, reverse electrodialysis, salinity gradient power, blue energy

## Abstract

The development of the most effective, suitable and economic ion-exchange membranes is crucial for reverse electrodialysis (RED)—the most widely studied process to harvest salinity gradient energy from mixing seawater and river water. RED utilizes two types of membranes as core elements, namely cation exchange membranes (CEM) and anion exchange membranes (AEM). Since the preparation of AEMs is more complex compared to CEMs, the design and development of anion exchange membranes have been the focus in this study. Homogeneous AEMs based on two types of polyepichlorohydrin (PECH) with different chlorine amounts (PECH-H, 37 wt% and PECH-C, 25 wt%) were synthesized, and first-time benchmarking of the membrane properties was conducted. In addition to physicochemical membrane properties, some instrumental analyses such as SEM, FTIR and DSC were investigated to characterize these anion-exchange membranes. Based on the results, although the PECH-H-type membrane had enhanced ion-exchange properties, PECH-C-based anion-exchange membranes exhibited a higher power density of 0.316 W/m^2^ in a lab-scale RED system. Evidently, there is room for the development of new types of PECH-C-based AEMs with great potential for energy generation in the RED process.

## 1. Introduction

Energy, water and food have represented three global issues faced by humankind for a long time. In particular, energy consumption and the search for alternative energy sources have become inevitable due to the depletion of fossil fuels and undesired climate change. One of the most interesting and probably the least-known renewable energy source is osmotic energy (or salinity gradient energy (SGE)). This type of energy, also called blue energy, has great potential worldwide since it is the sustainable energy extracted from the mixing of two water bodies with different salinities, such as river water and sea water [1,2,3,4,5].

There are now many techniques being developed to extract salinity-gradient energy and convert it into electrical energy, such as pressure-retarded osmosis (PRO), reverse electrodialysis (RED), and capacitive mixing (CAPMIX), which are commonly known [6,7,8]. However, other SGE technologies also exist, such as microbial RED, accumulator mediated mixing, hydrocratic generator, reverse vapor compression, and some other adsorption/desorption processes [9]. Among all those techniques, RED and CAPMIX utilize ion-exchange materials, and ion transport happens in an electrochemical manner. On the contrary, PRO uses a semipermeable osmotic membrane where the dissolved salts are retained and a solvent is allowed to pass, creating hydraulic pressure. RED is the most widely studied technique and has experienced sharp growth in recent years because of its unique advantages [2,7,10,11]. It is, for instance, the most efficient process when sea water and river water are considered as the feed, although there are applications wherein very-high-salinity solutions can also be used [12,13,14]. In addition, the use of treated wastewater effluents and other diverse applications of RED has contributed significantly to its versatility [10,15,16,17,18]. In contrast, CapMix utilizes the capacitive electrodes where the ion exchange membranes are not solely used [8,19]. This process is a relatively new technique based on the charging/discharging principle of electrodes and is still under development.

RED is an electrochemical process in which the ion-exchange membranes are used as core elements. It is basically the inverse of conventional electrodialysis (ED) [20,21], such that, in ED, energy is used to create a salinity difference, whereas energy is harvested in RED by the utilization of the two solutions with different salinities. In RED, membranes have a significant role such that the CEMs permit the transport of cations and AEMs permit the transport of anions from the high-saline compartment to the low-saline compartment, alternatingly mounted, forming a membrane stack [5,22,23]. Therefore, understanding the physicochemical properties of membranes and the development of those parameters determines the performance of not only the RED process but also other conventional ED-based processes [24,25,26]. Although the design of high-performance membranes is not the only parameter determining the RED performance, its role is huge among other factors such as the development of spacers, electrodes [27,28], stack-operation methods [10,29], and hybrid systems [30,31]. The design and development of AEMs are particularly challenging since multistage fabrication routes including toxic stages such as chloromethylation are conventionally employed. Thus, simpler routes for synthesis with reduced or eliminated toxic methods are becoming more attractive in terms of AEM preparation, specifically for RED applications.

Polyepichlorohydrin (PECH)-based AEMs have recently gained much attention because of single-step fabrication without the use of toxic chloromethylation reaction. In addition, simultaneous quaternization and crosslinking is possible when tertiary diamines are used to prepare these AEMs. Since the tailor-made AEMs for RED were first introduced in 2012 by Güler et al. [5], there have been several other works reported later on. Villafaña-López et al. [32] performed similar work on PECH-based AEM for RED, and the membranes were modified by glutaraldehyde and polyethyleneimine to improve membrane properties such as anionic selectivity and surface homogeneity. In another work, ultrathin PECH-based membranes were prepared specifically for RED by Jung et al. [33] by the use of spincoating on nanoporous alumina. Very recently, Reyes-Aguilera et al. used the electrospinning technique to prepare PECH membranes where the membrane morphology was varied with different electrospinning parameters [34]. These studies already presented some promising AEMs for RED applications; however, there is always room for development. The research should proceed towards the search for alternative polymers because correlations between different polymer materials and membrane characteristics have not been fully understood yet.

In this work, two types of PECH (polyepichlorohydrin (PECH-H) and polyepichlorohydrin-co-ethylene oxide (PECH-C))-based AEMs were particularly prepared for RED applications. For the first time, PECH-C-based AEMs were fabricated as promising alternatives as RED membranes. Simultaneous crosslinking and functionalization were performed without the use of toxic chloromethylation step. In particular, the chemical composition of AEMs synthesized and the correlations with the physicochemical properties were investigated. First-time benchmarking of these properties with the commercially available membrane has been also performed using the lab-scale RED system.

## 2. Materials and Methods

### 2.1. Materials

To fabricate the homogeneous AEMs, polyepichlorohydrin ((CH(CH_2_Cl)CH_2_O)_m_(CH_2_CH_2_O)_n_, EPICHLOMER C, 25 wt% chlorine and ((CH(CH_2_Cl)CH_2_O)_n_ H, 37 wt% Cl, Osaka Soda, Co., Ltd., Osaka, Japan), polyacrylonitrile, Mitsubishi Chemical Co. Ltd., Tokyo, Japan), 1,4-diazabicyclo [2.2.2] octane (Sigma Aldrich, Munich, Germany) were adopted. Dimethyl sulfoxide (Isolab) was used as the solvent for all the experiments.

Sodium sulfate, silver nitrate, and sodium chloride used for membrane characterization were obtained from Isolab (Wertheim, Germany) Potassium ferricyanide (K_3_[Fe(CN)_6_]) and potassium ferrocyanide (K_4_[Fe(CN)_6_]·3H_2_O) were provided from Proanalyst, Merck, Darmstadt, Germany. All chemicals were used as they were purchased from the suppliers.

### 2.2. Preparation of Homogeneous AEMs

**Figure 1:**
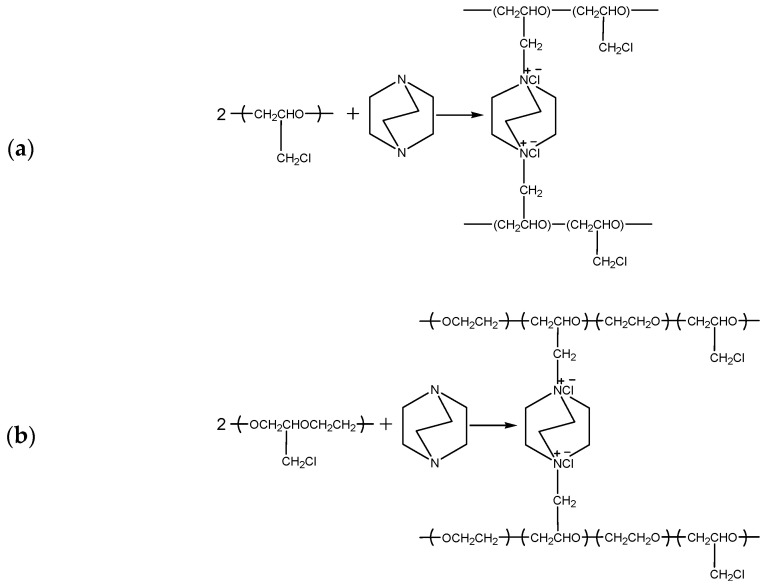
Reaction mechanisms of simultaneous amination and crosslinking of (**a**) PECH-H and (**b**) PECH-C type polymers.

Anion-exchange membranes (AEMs) with homogeneous bulk structures were fabricated by solution-casting. This was later followed by solvent evaporation as reported in our previous work [5]. The amination and crosslinking reactions were performed simultaneously according to the schemes in Figure 1. For this purpose, membrane cast solution was prepared by mixing three constituents, polyepichlorohydrin (PECH), polyacrylonitrile (PAN) and 1,4-diazabicyclo [2.2.2] octane (DABCO). Then, solutions of PECH-H or PECH-C (15 wt%), PAN (12 wt%), and DABCO (12.25 wt%) were prepared. These solutions were then mixed in a flask for half an hour at 80 °C to obtain a clear casting solution. Membrane solution was cast onto a glass plate which was sealed with a glass cover. This membrane cast solution was positioned in a convection oven at 110 °C for 2 h. After that, the sealing was opened, and the remaining solvent was further evaporated at 130 °C for 30 min. Membrane samples were later immersed in 0.1 M NaCl solution after cooling down to room temperature (20 °C) and stored in the same solution continuously until further use.

In order to prepare different sets of membranes with different compositions, two terms, namely the blend ratio (BR) and excess diamine ratio (EDR), were defined to investigate the effects of concentration of active polymer PECH with respect to inert polymer PAN, and the concentration of diamine DABCO with respect to chloromethyl functional groups in active-polymer PECH, respectively [5].

### 2.3. Membrane Characterization

#### 2.3.1. Thickness of the PECH Membranes

After the removal of surface water of the PECH membrane using blotting paper, the film thickness was immediately measured using a precise micrometer (Mitutoyo Co., Kawasaki, Japan) at various regions of the membrane, and the average thickness was determined.

#### 2.3.2. Scanning Electron Microscopy

To investigate the morphology of membrane films, QUANTA 400F Field Emission SEM (Eindhoven, The Netherlands) was used. Membrane samples were placed on the holders and coated with gold to provide conductivity. The surface and cross-section of the membrane samples were investigated.

#### 2.3.3. Fourier Transform Infrared Spectroscopy

The surface characterization of the prepared dry PECH membrane samples were performed by Fourier transform infrared (FTIR) spectroscopy (Thermo Scientific-Nicolet 510, Madison, WI, USA). Attenuated total reflectance (ATR) mode was used during the analyses. The average of 32 scans was taken for the spectrum with a resolution of 4 cm^−1^ at wavelengths between 4000 and 400 cm^−1^.

#### 2.3.4. Ion Exchange Capacity

The ion exchange capacity (IEC) is defined as the amount of fixed-charge groups per unit weight of dry polymer. Initially, membranes were immersed in 3 M NaCl solution for 15 h at room temperature (20 °C). Then, Milli-Q water was used to rinse membranes. After that, the membranes were exposed to 1.5 M Na_2_SO_4_ for 3 h. To calculate the IEC, Na_2_SO_4_ solution was back-titrated with a 0.1 M AgNO_3_ solution [35]. Lastly, membranes were dried at 30 °C in a vacuum oven until constant weight was reached. The IEC was determined via Equation (1).

(1) $$\mathrm{IEC}=\frac{V_{\mathrm{AgNO}3}}{m_{\mathrm{dry}}}\times C_{\mathrm{AgNO}3}$$

where $V_{\mathrm{AgNO}3}$ and $C_{\mathrm{AgNO}3}$ are the volume and concentration of the AgNO_3_ solution, respectively, while $m_{\mathrm{dry}}$ represents the weight of dry membrane samples.

#### 2.3.5. Swelling Degree

The swelling degree (SD) is a physicochemical parameter that directly determines the mechanical properties of a membrane. To determine the SD, prepared anion-exchange membranes were soaked into demineralized water for 24 h. After weighing the wet membranes, they were dried until a constant weight was reached at approximately 30 °C. After measuring the dry membranes, the swelling degree was calculated using Equation (2):

(2) $$\mathrm{SD}=\frac{m_{\mathrm{wet}-}m_{\mathrm{dry}}}{m_{\mathrm{dry}}}\times 100\%$$

where $m_{\mathrm{wet}}$ and $m_{\mathrm{dry}}$ represent the weight of membrane samples in wet and dry states, respectively.

#### 2.3.6. Fixed Charge Density

The fixed charge density (C_fix_), which can be defined as the amount of ion exchanging functional groups per water content in the membrane, has an impact on the ion transport properties of the membranes. C_fix_ can be calculated using the ratio of IEC and SD values of the prepared membranes as defined in Equation (3) [35].

(3) $$C_{\mathrm{fix}}=\frac{\mathrm{IEC}}{\mathrm{SD}}$$

#### 2.3.7. Differential Scanning Calorimetry

To observe the morphology of different PECH-type polymers and their crystallinity behavior, differential scanning calorimetry (DSC) analyses were performed. The melting temperature (T_m_) and heat of fusion ((ΔH_fusion_) of the quaternized and pristine membranes were measured using Perkin Elmer (Diamond DSC) differential scanning calorimetry in the temperature range of −70 to 200 °C at a scanning rate of 20°/min under a N_2_ atmosphere.

### 2.4. RED Tests

#### 2.4.1. The RED System

**Figure 2:**
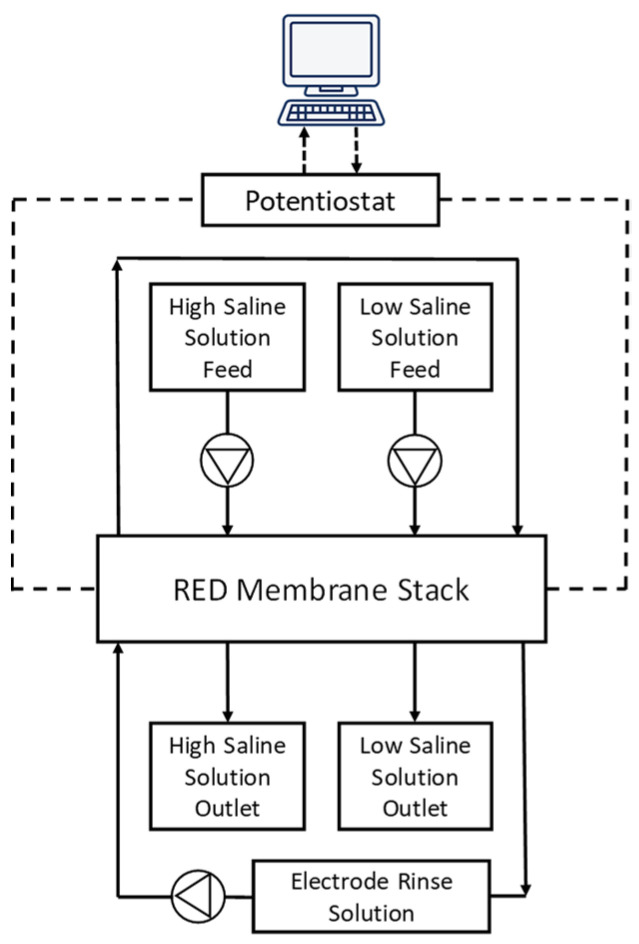
Flow diagram of the RED system.

Custom-made AEMs (PECH-C and PECH-H) were fabricated to be used in the lab-scale RED system with a 10 cm × 10 cm electrode area (STT Products B.V., BA Schiedam, The Netherlands). The RED stack was formed by installing three PECH-based AEMs and four Neosepta CMX cation-exchange membranes alternatingly mounted between a reversible anode and cathode (Ru-Ir oxide-coated Ti mesh with 10 cm × 10 cm active area, Magneto Special Anodes BV, BA Schiedam, The Netherlands). The intermembrane thickness was kept fixed at 400 µm using Nitex polyamide woven spacers. An electrode rinse solution was circulated containing the mixture of three solutions (0.25 M NaCl, 0.05 M K_4_Fe(CN)_6_ and 0.05 M K_3_Fe(CN)_6_) at 300 mL/min. Feed waters (0.507 M NaCl as artificial seawater and 0.017 M NaCl as artificial river water) were fed to the stack with peristaltic pumps at several flow rates between 30 and 120 mL/min at room temperature (20 °C). A schematic flow diagram of the lab-scale RED system is given in Figure 2.

#### 2.4.2. Electrochemical Measurements

The RED performance of the produced AEMs was investigated using a potentiostat (Gamry Instruments Reference 3000, Warminster, PA, USA) in chronopotentiometry mode. Up to 40 A/m^2^ of current steps were continued for 30 s. The maximum power density was calculated for each feed flowrate by the multiplication of voltage and current. The maximum power density was then corrected by subtracting the power output of a separate run, so-called a blank test, using only one CEM in the stack. Power density (W/m^2^) is evaluated by dividing this power output by the total membrane area.

## 3. Results and Discussion

### 3.1. Membrane Thickness and Morphology

In order to attain sufficient film-forming properties and to make benchmarking with commercial AEMs, PECH-based membranes with about 150 µm were fabricated in this work. Many commercial ion-exchange membranes for RED, ED, EDI, or other electromembrane processes have about a 100–150 µm film thickness (e.g., Neosepta, Selemion), although a few have thicker (e.g., Ralex) and some have thinner film thicknesses (e.g., FumaTech) [36].

**Figure 3:**
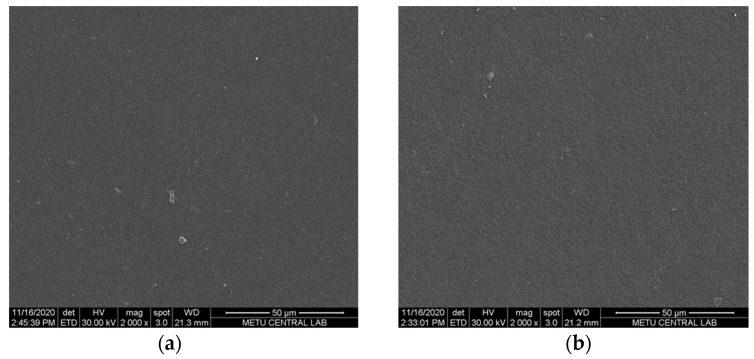
Surface morphology of (**a**) PECH-H and (**b**) PECH-C membranes (magnification: 2000×).

**Figure 4:**
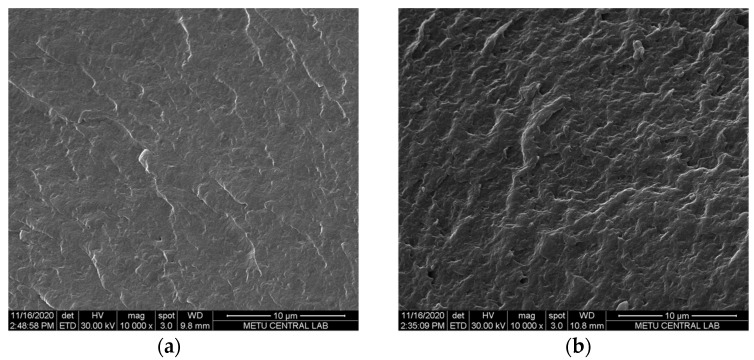
Morphology of the cross-section of (**a**) PECH-H and (**b**) PECH-C type membranes (magnification: 10,000×).

To examine the anion-exchange membranes’ morphology on both the surface and cross-section, SEM analysis was performed at a magnification of 2000×. Figure 3 and Figure 4 show the morphology of surface and cross-section of the prepared membranes.

In the RED system, membranes with a dense bulk structure are preferred to prevent or reduce the water transport through the membranes. According to the SEM images, both the surface and cross-section morphology of PECH-H- and –C-type membranes are very similar (Figure 3 and Figure 4). Even at high magnification (×10,000), this similarity is observed at the cross-section of the membranes (Figure 4). In all prepared AEMs, a non-porous polymeric structure was obtained as it is predictable due to the solvent evaporation technique used to fabricate these membranes. These membranes also have the flexibility to be prepared by filling the pores of a porous substrate forming a pore-filling membrane with a very thin film thickness. However, this is out of the scope of this work.

### 3.2. FTIR Analysis

**Figure 5:**
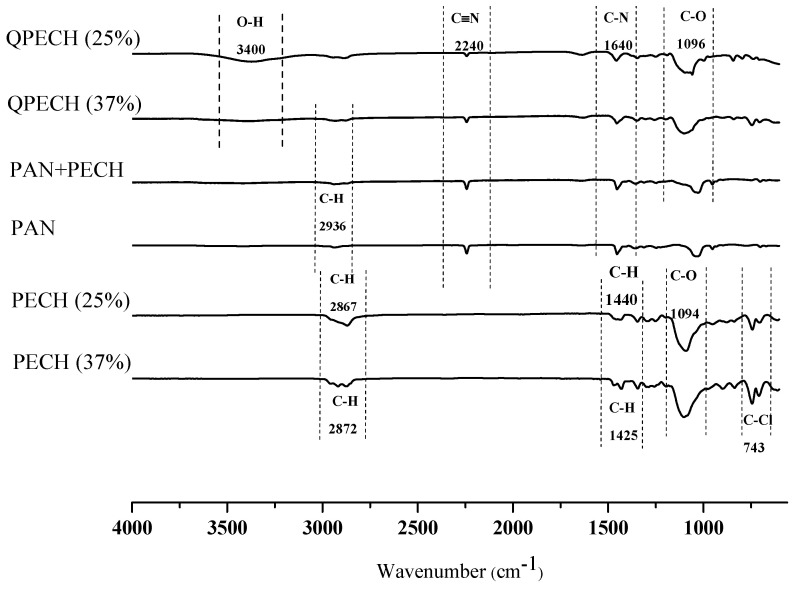
FTIR spectra of the polymers.

To verify quaternization (i.e., amination) of the active PECH polymer with DABCO, FTIR analysis was performed. Figure 5 shows the FTIR spectra of pristine PECH polymers, PAN, PECH and PAN blend before quaternization and aminated PECH (QPECH)/PAN, respectively.

In Figure 5, characteristic peaks of PECH and PAN are observed. Stretching vibration bands of –C-H groups were detected in the region at 2687 cm^−1^ and 2872 cm^−1^. At wavenumbers of 1425 cm^−1^ and 1440 cm^−1^, scissoring bands of –CH_2_ groups were observed. The wavenumber 743 represents C-Cl bondings of the functional groups of PECH polymers. On the other hand, a -C≡N bond of pristine PAN was visible at peak 2240 cm^−1^. The same peak was still visible at the blend of PECH and PAN polymers. The peak at 3400 cm^−1^ represented O-H stretches, indicating free water. When the active polymer is aminated with DABCO, C-N bonds appeared at 1640 cm^−1^, showing successful quaternization of all membranes. Nevertheless, the quaternized PECH-C-type membrane had more intense peaks, which can be estimated to have a relatively higher amount of functional groups [32,35]. In addition, O-H stretching is enhanced in PECH-C, which also supports this interpretation.

### 3.3. Effect of Blend Ratio on Membrane Characteristics

**Figure 6:**
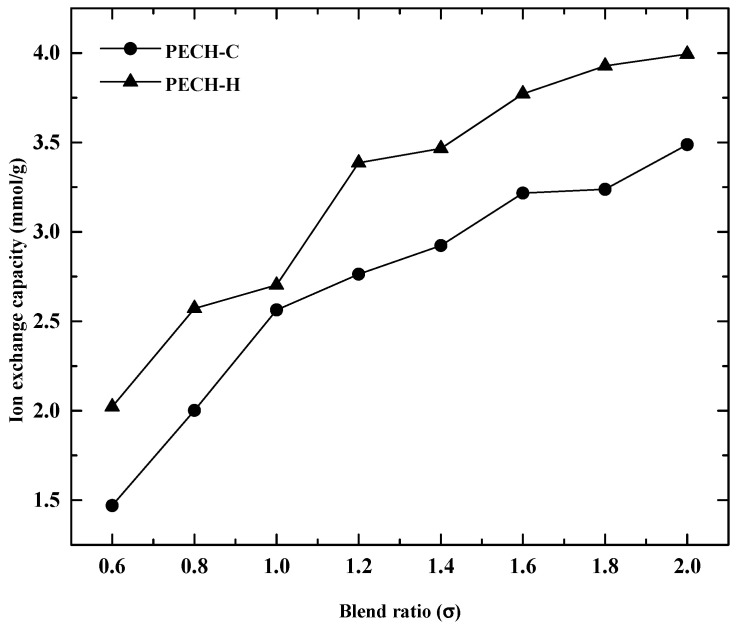
Impact of blend ratio on ion exchange capacity for the PECH membranes at EDR of 2.

**Figure 7:**
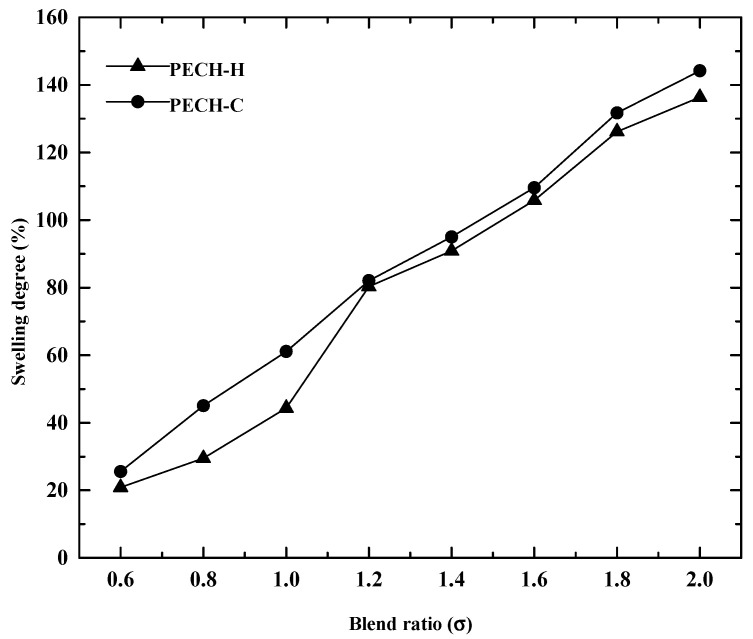
Impact of blend ratio on swelling degree for the PECH membranes at EDR of 2.

Differently from our previous work, the effects of PAN-PECH blending on membrane properties were investigated at various blend ratios between 0.6 and 2. In addition, to determine the optimized PECH-based AEM for RED application, two types of PECH were employed: one has a homopolymer of epichlorohydrin, (PECH-H) and the other has the copolymers of epichlorohydrin and ethylene oxide, (PECH-C). The effect of blending PECH and PAN on IEC and SD for the prepared membranes was shown in Figure 6 and Figure 7.

For both types of PECH membranes, IEC and SD increased with an increasing blend ratio. High values of IEC and SD were observed up to 4.0 mmol/g and 140%, respectively. These results are in the same direction with the data reported by Güler et al. [5], where similar behavior of these properties was realized such that up to IEC of 3.0 mmol/g and SD of 120% were observed for PECH-H membranes. An increase in the concentration of active polymer leads to an increase in the active sites of the AEMs. In other words, charged groups in a polymer chain increases with an increase in the concentration of active polymer, resulting in increasing IEC [10,37]. On the other hand, the enhancement of IEC promotes an increase in swelling, which is undesirable up to a certain level. Overall, PECH-H-type AEMs are more advantageous than PECH-C in terms of having a high level of IEC.

According to Figure 7, PECH-C-based AEMs exhibited higher SD. That means more water uptake of PECH-C-type membranes were noticed than that of PECH-H at all blend ratios, although it is not significant at values higher than 1.2.

### 3.4. Effect of Excess Diamine Ratio on Membrane Characteristics

**Figure 8:**
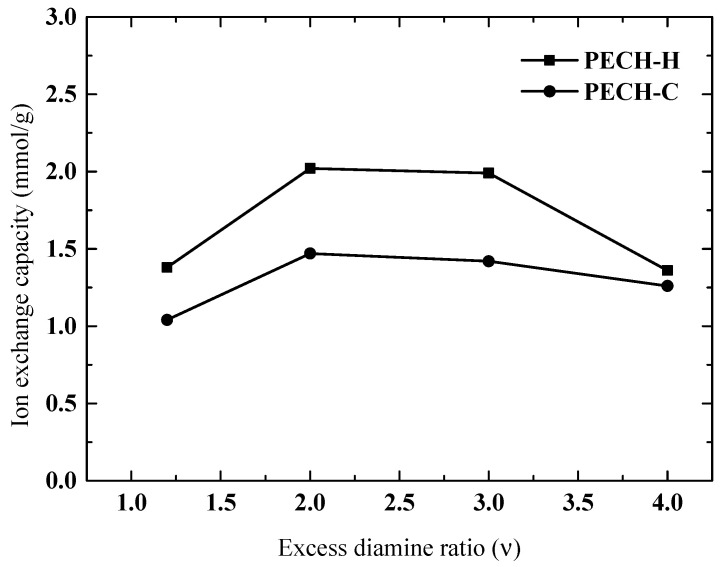
Effect of excess diamine ratio on ion exchange capacity for both types of PECH membranes at BR of 0.6.

**Figure 9:**
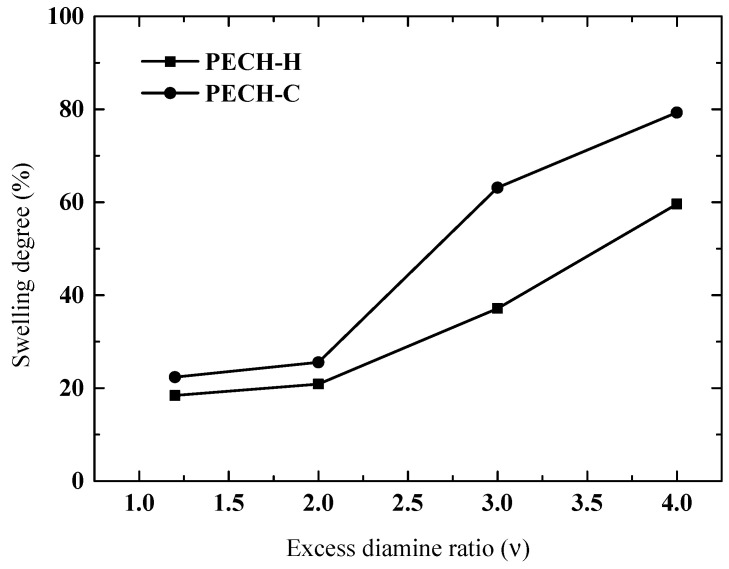
Effect of excess diamine ratio on swelling degree for both types of PECH membrane at BR= 0.8.

The effect of excess diamine ratio (EDR) on SD and IEC is investigated between 1.2 and 4.0 (Figure 8 and Figure 9). In our previous work, EDR values higher than 4 had an insignificant impact on IEC and SD [5]. This time, 0.6 was chosen as the fixed-blend ratio (BR) for this parametric work, resulting in the lowest SD values, although the effect of EDR may show different trends at various BR.

In Figure 8, the ion exchange capacity exhibits an increase up to an EDR of 2, and afterwards, a slight leveling off is observed for both types of PECH membranes. It may give the impression that bi-quaternization (i.e., attachment of DABCO at both ends to the PECH polymer chains) is effective up to an EDR of 2. After that value of EDR, the SD continuously increases, indicating that crosslinking is not effective at controlling the excessive water uptake of the membranes. That is why it was chosen to investigate the effect of BR at this EDR value as reported in the previous section. Again, in this parametric study on EDR, PECH-C-type membranes exhibited higher IEC and SD properties.

### 3.5. DSC Analyses and Impact of Crystallinity

As the polymer structures of PECH-H- and -C-type membranes are different, this can be confirmed by investigating the morphology by differential scanning calorimetry (DSC) analyses. The impact of differences in the alignment of molecular chains (i.e., crystallization) on the ion-exchange capability of the membranes can also be investigated by these analyses.

There are some vital parameters affecting the crystal structure of the homogenous membranes such as enthalpy of fusion (ΔH_fusion_), melting temperature (T_m_), chain flexibility and interactions. These factors also have a significant impact on the mechanical properties [38]. Nevertheless, the mechanical properties but also the ionic selectivity of membranes are influenced by crystallinity because crystal regions are able to function as polymer crosslinks. As ion transport appears easily in amorphous regions, crystal regions have supremacy on the ion selectivity of the polymer. In other words, permeability decreases with the increase in crystalline structure and molecular orientation because of the decrease in diffusion [39]. Consequently, crystal regions inhibit the effectiveness of the transporting materials.

**Figure 10:**
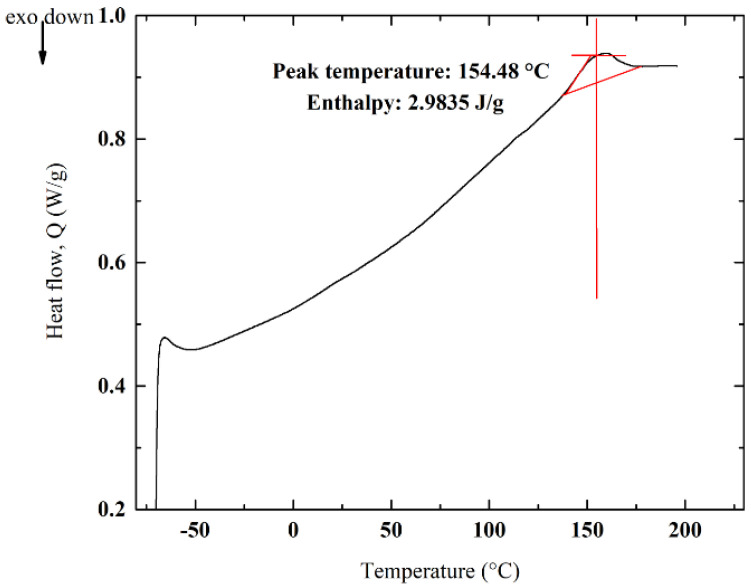
DSC curves for PECH-H in N_2_.

**Figure 11:**
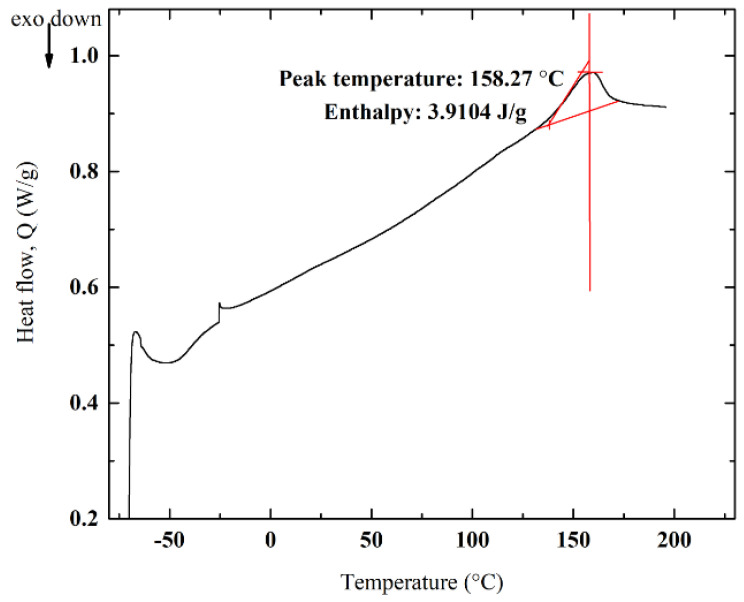
DSC curves for PECH-C in N_2_.

DSC analysis was implemented to determine the Tm of the prepared membranes. These values regarding PECH-H- and –C-type membranes were shown in Figure 10 and Figure 11, respectively.

In DSC analysis, both crystalline and semi-crystalline polymers melt when heated. Highly crystalline polymers require more energy to break or vibrate the polymeric chains. Therefore, an interpretation of the direct correlation between the melting point and crystallization can be made [38]. Owing to the higher T_m_ (158.27 °C) and ΔH_fusion_ (3.9104 J/g) of PECH-C than T_m_ (154.48 °C) and ΔH_fusion_ (2.9835 J/g) of PECH-H, PECH-C-type membranes have more a crystalline character (Figure 10 and Figure 11). Crystalline regions restrict the diffusion of ions, whereas ions can pass through amorphous regions. For instance, this phenomena resembles the one such that the permeability of crystalline poly(vinyl alcohol) and polyamide (Nylon 6) is very low, while amorphous polydimethylsiloxane has higher permeance of species [40]. According to Figure 11, PECH-C-type AEM has elevated T_m_, explaining its more regular structure (i.e., crystalline). Thus, anion (e.g., chlorine ions) transport through the membrane is reduced, and it yields a low ion-exchange capacity.

### 3.6. Benchmarking of Membrane Properties

To summarize the properties of the prepared membranes and make benchmarking with commercial counterparts, the data are shown in Table 1. These membranes are also chosen to be tested to determine their performances in the RED system. We found that the membrane thicknesses are comparable as they are in the range of 120–160 µm. When PECH-type membranes are compared, it is observed that the PECH-C-type membrane has a lower IEC but slightly higher SD than that of the PECH-H-type membrane. This may be attributed to the effectiveness of crosslinking in PECH-H-type membranes as DABCO has the capability of quaternization and crosslinking at the same time. Since the water uptake is better controlled in PECH-H type-membranes, the fixed charge density becomes higher. The fixed charge density is a parameter that affects other membrane properties such as permselectivity and electrical resistance, which were not determined in this work. In our previous work, these parameters were extensively investigated such that the permselectivity and area resistance increase in PECH-based membranes when there is an increase in fixed charge density [5]. In general, the physicochemical properties of custom-made PECH-based membranes are comparable with the commercially available ones.

### 3.7. RED Performance

**Figure 12:**
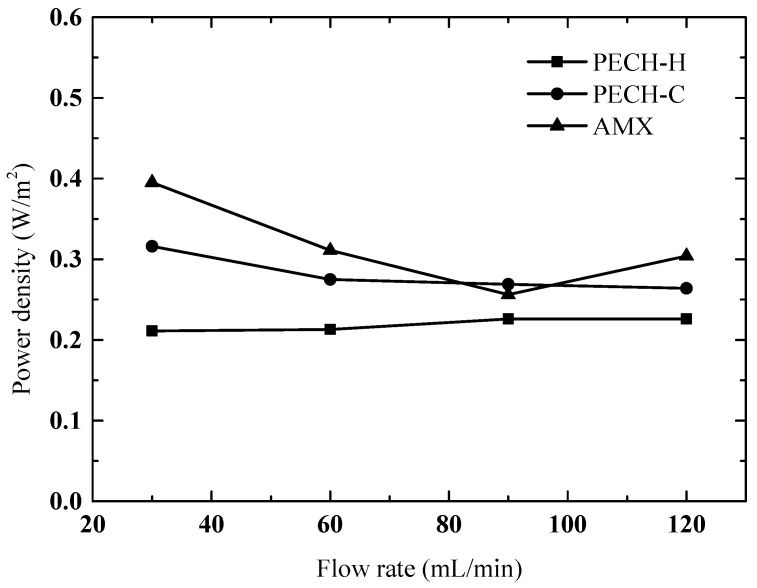
Benchmarking of RED performance of the two types of PECH AEMs with commercial Neosepta AMX membrane (CEM: Neosepta CMX).

Here, for the first time, we report the performance of ethylene-oxide containing PECH anion-exchange membranes (PECH-C) in the RED system. Their performance is compared to commercial counterparts. All the membranes were prepared with a similar film thickness, having a BR of 0.6 and EDR of 2.0 (Table 1). The RED stack was built with either commercial Neosepta CMX and AMX membranes, or custom-made PECH membranes as AEMs coupled with Neosepta CMX as CEMs. Since a relatively large intermembrane distance (400 µm) was used, the feed flow rate did not have a significant impact on the power output (Figure 12). Even a slight decrease may be observed at higher flow rates, resulting in lower residence time, which is not enough for sufficient ion transport through the membranes. That is also the case when the internal resistance due to low saline solution compartment is comparatively high. Nevertheless, the RED setup was operated properly to allow us to make a fair performance evaluation. In Figure 12, it was shown that the energy-generating performances of PECH-C and AMX membranes are similar, whereas the one of PECH-H is lower. These findings are in the same direction that the physicochemical properties of PECH-C and Neosepta AMX membranes are similar, as shown in Table 1. In addition, the fixed charge density of PECH-H is the highest by allowing a high area resistance, as indicated in our previous work, implying that a high fixed charge density may cause a reduction in power density [32,35]. In fact, it is not reliable to make a direct performance comparison between this work and previous studies due to differences in some parameters used, such as the film thickness of membranes and intermembrane distance. Nevertheless, for instance, PECH-H-type membranes exhibited a 12% reduction in power density when the fixed charge density increased by 20% [5]. In this work, PECH-H membranes had 70% higher fixed charge density and exhibited 14% lower power density than PECH-C-type membranes. Therefore, it can be reported that the best-performing membrane, PECH-C, with a BR of 0.6 and EDR of 2.0, can produce a power density of up to 0.32 W/m^2^. It is worth mentioning that it is possible to make these membranes as thin as 100 µm and even thinner according to our past experience. By ensuring this, we expect a superior RED performance compared to other commercially available alternatives.

## 4. Conclusions

For the first time, we have shown a comparison between PECH-based membranes containing ethylene oxide copolymer (PECH-C) and the other membranes based on RED performance. These membranes were made via a single-step quaternization-crosslinking method, which represents a highly promising alternative for custom-made RED membranes. The highest power density produced by PECH-C-type membranes coupled with Neosepta CMX was 0.32 W/m^2^, whereas it was 0.39 W/m^2^ for Neosepta AMX coupled with Neosepta CMX at the same operating conditions. However, there is always some room for development of PECH-based membranes, which will be always an attractive option for RED processes because of its single-step fabrication without toxic reactions such as chloromethylation.

## Figures and Tables

**Table 1:** Properties of custom-made and commercial membranes (custom-made PECH membranes: BR = 0.6 and EDR = 2.0).

Membrane	Thickness (µm)	IEC (mmol/g)	SD (%)	C_fix_ (mmol/g H_2_O)
PECH-H AEM	154	2.02	20.88	9.70
PECH-C AEM	160	1.47	25.56	5.70
Neosepta AMX	134	1.40	26.00	5.40
Neosepta CMX	158	1.62	18.00	9.00

## Data Availability

The data available in this study are available on request from the corresponding author.
